# Natural Compounds Modulating Mitochondrial Functions

**DOI:** 10.1155/2015/527209

**Published:** 2015-06-18

**Authors:** Lara Gibellini, Elena Bianchini, Sara De Biasi, Milena Nasi, Andrea Cossarizza, Marcello Pinti

**Affiliations:** ^1^Department of Surgery, Medicine, Dentistry and Morphological Sciences, University of Modena and Reggio Emilia, Via G. Campi 287, 41125 Modena, Italy; ^2^Department of Life Sciences, University of Modena and Reggio Emilia, Via G. Campi 287, 41125 Modena, Italy

## Abstract

Mitochondria are organelles responsible for several crucial cell functions, including respiration, oxidative phosphorylation, and regulation of apoptosis; they are also the main intracellular source of reactive oxygen species (ROS). In the last years, a particular interest has been devoted to studying the effects on mitochondria of natural compounds of vegetal origin, quercetin (Qu), resveratrol (RSV), and curcumin (Cur) being the most studied molecules. All these natural compounds modulate mitochondrial functions by inhibiting organelle enzymes or metabolic pathways (such as oxidative phosphorylation), by altering the production of mitochondrial ROS and by modulating the activity of transcription factors which regulate the expression of mitochondrial proteins. While Qu displays both pro- and antioxidant activities, RSV and Cur are strong antioxidant, as they efficiently scavenge mitochondrial ROS and upregulate antioxidant transcriptional programmes in cells. All the three compounds display a proapoptotic activity, mediated by the capability to directly cause the release of cytochrome c from mitochondria or indirectly by upregulating the expression of proapoptotic proteins of Bcl-2 family and downregulating antiapoptotic proteins. Interestingly, these effects are particularly evident on proliferating cancer cells and can have important therapeutic implications.

## 1. Introduction

Mitochondria are unique membrane-enclosed organelles found in eukaryotic cells; they are usually described as the “powerhouse” of the cell as they contain the molecular machinery that governs many distinct metabolic pathways taking place within these organelles, including (but not limited to) pyruvate oxidation, fatty acid *β*-oxidation, Krebs cycle, and oxidative phosphorylation (OXPHOS) [1]. Mitochondria importance is not limited to cell metabolism or regulation of bioenergetics pathways. Indeed, during the last decades, their role as master regulators controlling stress responses and cell death has emerged [2–4]. Furthermore, mitochondria are the main intracellular source of reactive oxygen species (ROS) [5]. The multiple functions of mitochondria have more and more underlined the great relevance of such organelle in biomedicine. Indeed, not only are they responsible for several genetic diseases, due to inherited mutations of mitochondrial DNA (mtDNA), but also they play a main role in the processes of inflammation, aging, cancerogenesis, and neurodegeneration [3, 6–11].

In the last decades, a particular interest has been devoted to studying the effects of natural compounds of vegetal origin (often referred to as phytochemicals, herbals, or phytocompounds) on human cells, as these compounds are often taken with the diet at biologically active concentrations and constitute fundamental components of traditional medicine of several countries [8, 12–18]. Many of these compounds turned out to exert their functions by affecting mitochondrial functions, either directly, by inhibiting specific enzymes, or indirectly, by modulating signal from or to mitochondria [19–23].

**Figure 1:**
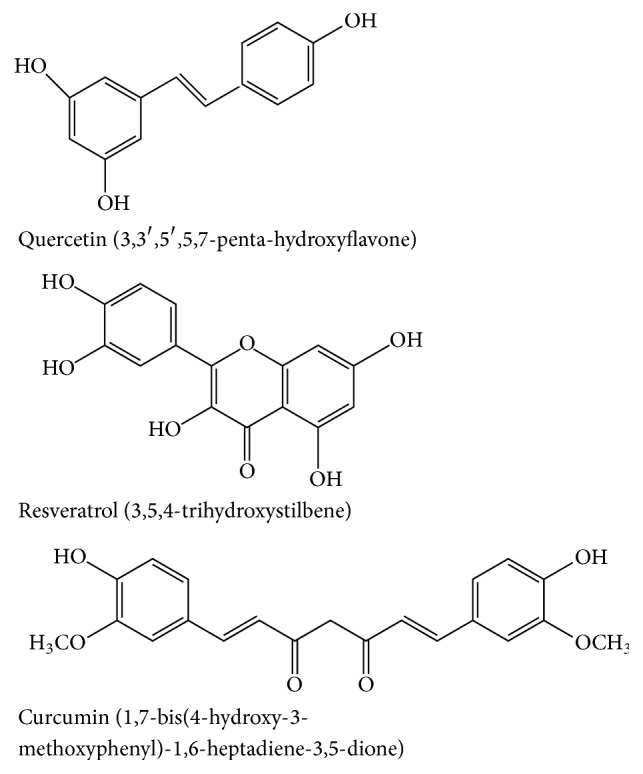
Chemical structure of quercetin (Qu), resveratrol (RSV), and curcumin (Cur).

In this review, we will discuss recent discoveries concerning the effects of natural compounds on mitochondria, with a major emphasis on resveratrol (RSV), the flavonoid quercetin (Qu), and curcumin (Cur) derivatives, probably the most studied plant-derived natural compounds (Figure 1). Resveratrol (3,5,4-trihydroxystilbene) is a stilbenoid naturally produced by several plants in response to environmental stress or injury and present in many fresh fruits (including grapes, blueberries, and raspberries) or fruit-derived foods. Quercetin (3,3′,4′,5,7-pentahydroxyflavone) is a main dietary flavonoid, present in vegetables, fruits, seeds, nuts, tea, and red wine [11, 24, 25]. Curcumin (1,7-bis(4-hydroxy-3-methoxyphenyl)-1,6-heptadiene-3,5-dione) is a diarylheptanoid derived from the rhizome of* Curcuma longa*, which exhibits cancer growth inhibition both* in vitro* and* in vivo* [26, 27], by suppressing cell proliferation and inhibiting tumourigenesis [28–33].

## 2. Mitochondria, Oxidative Phosphorylation, and Natural Compounds

Mitochondria are the organelle where cell respiration, OXPHOS, and synthesis of most cellular ATP take place. Since these metabolic processes involve dozens of proteins or protein complexes, effects of phytochemicals on them are very complex and often difficult to interpret and are subject of intensive investigation. ATP is synthesized in mitochondria by F0F1 ATP synthase, a multimeric complex consisting of the catalytic F1 sector (a3b3cde) and the trans-membrane proton pathway, the F0 sector (ab2c10). Several phytochemicals, including piceatannol, Qu, RSV, Cur, (−)epigallocatechin gallate, (−)epicatechin gallate, curcumin, genistein, or biochanin, are able to inhibit F0F1 ATPase, both in mitochondria of mammalian cells or in prokaryotic cells [19, 22, 23, 34, 35].

### 2.1. Effects of Quercetin on Oxidative Phosphorylation

The effects of Qu on mitochondrial biochemical pathways are of particular interest, since Qu can specifically accumulate in these organelles [36]. More than 40 years ago it was shown that Qu inhibits mitochondrial ATP synthase, similarly to well-known inhibitors of mitochondrial electron transport. Moreover, Qu strongly affects the succinate oxidase as well as the NADH oxidase activities but has no effect on OXPHOS in submitochondrial particles [37]. More recently, it has been shown that Qu can uncouple OXPHOS at concentrations as high as 30 *μ*M. Interestingly, at concentration >50 *μ*M, Qu stimulates oxygen consumption, inhibits OXPHOS, decreases mitochondrial membrane potential, and causes Ca^2+^ release [38, 39]; the uncoupling effect, with a dose-dependent stimulation of State 2 respiration rate, has also been observed in rat heart mitochondria [40].

### 2.2. Effects of Resveratrol on Oxidative Phosphorylation

Resveratrol improves mitochondrial function by inducing the expression of genes for oxidative phosphorylation and mitochondrial biogenesis; this effect is mediated by a decrease in the acetylation of PGC-1alpha—one of the master regulators of mitochondrial biogenesis—and by the subsequent increase in its functional activity [41]. Several studies performed* in vivo* on rats further demonstrated the beneficial effect of RSV on mitochondria. In particular, dietary supplementation with RSV causes an amelioration of several mitochondrial functions (oxygen consumption, activity of respiratory enzymes, and activity of lipid-oxidizing enzymes) [42–44]. It must be noted, however, that in mitochondria isolated from rat brain RSV inhibits the mitochondrial F0F1-ATPase activity in a concentration-dependent manner, in the range of 0.7–70 *μ*m, suggesting that RSV can also impair mitochondrial metabolic pathways [23].

### 2.3. Effects of Curcumin on Oxidative Phosphorylation

In isolated mitochondria from rat liver, Cur acts as a protonophoric uncoupler [45]. In this model, Cur decreases ATP biosynthesis, activates F0F1-ATPase in a dose-dependent manner (a common feature of protonophoric uncouplers), and inhibits respiration at concentrations >50 *μ*M [45]. However, it is interesting to note that Cur inhibits the F0F1-ATPase in rat brain mitochondria, indicating that different—in this case, opposite—effects of this phytochemical can be observed in the same organelle from different tissues [23, 45]. A possible mechanism of this action has been elucidated in* Escherichia coli*, where Cur directly inhibits F1 ATPase activity by disrupting the beta subunit catalytic site conformational transitions [22, 34].

## 3. Mitochondria, Reactive Oxygen Species, and Natural Compounds

Quantitative date on isolated mitochondria indicates that up to 5% of oxygen consumption is due to superoxide anion (O_2_^∙−^) generation [46]. However, superoxide generation is heavily influenced by the cell type and by the respiration steady state; under physiologic conditions, the superoxide production is estimated to be about 0.1% of the respiratory rate [47]. Mitochondrial ROS are not just dangerous molecules: they also regulate several cell processes, including (but not limited to) apoptosis, autophagy, and unfolded protein response [5]. Quercetin, resveratrol, and curcumin can modulate in several ways the levels of different ROS and other free radicals within the cell (Table 1). Nevertheless, it must be noted that the capability of phytochemicals to directly scavenge ROS is probably not very relevant* in vivo* as, at the concentrations that they can reach within the cell, their scavenging effect is marginal if compared with detoxifying systems such as GSH. However, these compounds can indirectly exert an antioxidant activity by modulating antioxidant cell response—an effect that is much more important* in vivo*.

### 3.1. Effects of Quercetin on Mitochondrial ROS

Quercetin can exert both antioxidant and prooxidant activity [11]. Because of the high number of hydroxyl groups and conjugated *π* orbitals, Qu can efficiently scavenge mitochondrial ROS such as O_2_^∙−^ and hydrogen peroxide (H_2_O_2_) [48]. The reaction of Qu with O_2_^∙−^ leads to the generation of the semiquinone radical and H_2_O_2_. Then, Qu reacts with H_2_O_2_ and decreases its levels in the presence of peroxidases [49]. During the same process, potentially harmful reactive oxidation products can also be formed: semiquinone radical, the first product of Qu, is unstable and undergoes a second oxidation reaction that produces Qu-quinone, a molecule capable of damaging DNA and causing lipid peroxidation [50].

Qu can alter ROS metabolism by directly lowering the intracellular pool of GSH [51–53]. Indeed, Qu reacts with ROS and forms semiquinone and quinone radicals [49], which are highly reactive toward thiols, and preferentially react with GSH [54]. Thus, Qu depletes GSH in a concentration-dependent manner [54]. This phenomenon has been observed not only in cell lines, but also* ex vivo*: in isolated rat liver nuclei, Qu reduces, in a dose-dependent manner, nuclear GSH content [55]. Finally, Qu can indirectly affect intracellular ROS levels by inhibiting enzymes related with antioxidant activity, such as thioredoxin reductase and the glutathione S-transferase (GST) activity [55, 56].

Qu can also modulate the antioxidant pathway triggered by nuclear factor-erythroid 2 related factor 2 (Nrf2, a master regulator of antioxidant response). In normal conditions, Nrf2 is bound to Keap-1, which represses its activity by targeting it for ubiquitin degradation pathway [57, 58]. In the presence of oxidative stress, NRf2 is released from Keap-1 and translocates into the nucleus, where it activates the antioxidant transcriptional programme; this leads to the upregulation of genes involved, at least in part, in the increase of cell glutathione content. In HepG2 cells, Qu at the dose of 50 uM is able to rapidly (within 60 minutes) induce the phosphorylation and translocation into the nucleus of Nrf2, to later inhibit both effects. This activation is correlated with the activation of the GSH-related antioxidant/detoxifying enzymes [59]. In longer exposition, Qu causes the increase of Nrf2 levels by increasing its transcription, and by stabilizing the protein through the inhibition of its ubiquitination and degradation. Furthermore, Qu is able to decrease the levels of Keap-1, the inhibitor of Nrf2, at the posttranslational level. Thus, the higher levels of Nrf2 determine an increase in the activity of the Nrf2-dependent antioxidant-responsive element/electrophile-responsive element (ARE/EpRE) and the transcription of a series of genes involved in antioxidant response, such as the NADPH:quinone oxidoreductase (NQO1) [60].

Studies performed* in vivo* on rats have shown that Qu can have a protecting role in ischemia/reperfusion injury in different brain and heart cell types, by attenuating the cytotoxic effects of ROS and decreasing mitochondria-mediated apoptosis [61–63].

### 3.2. Effects of Resveratrol on Mitochondrial ROS

The antioxidant activity of RSV has been shown by a considerable number of reports and observed in transformed cells of different origin, as well as in nontransformed cells. Resveratrol decreases ROS in mitochondria as it acts as a potent scavenger of superoxide anion, hydrogen peroxide, and hydroxyl radical (OH^*∙*^), inhibits lipid peroxidation, and helps to replenish glutathione levels [64–67]; the antioxidant activity of RSV results in a cytoprotective effect on several cell types, including (but not limited to) keratinocytes, cardiomyocytes, adipocytes, neurons, and brain tissue [67–74].

Resveratrol can also exert its antioxidant activity in an indirect manner, by modulating the expression of mitochondrial proteins or by increasing the expression of ROS scavenging enzymes. As in the case of Qu, RSV is able, in a dose-dependent manner, to activate the antioxidant pathway triggered by Nrf2 in keratinocytes and in cultured coronary arterial endothelial cells [73, 75]. Also in this case, Nrf2 activation determines a higher ARE activity and a significant upregulation of Nrf2 target genes, such as NQO1 and HO-1 [75].

In endothelial cells, RSV reduces mitochondrial ROS generation by increasing SIRT3 levels within the mitochondria, which in turns leads to the to increased complex I activity and ATP synthesis through the upregulation of mitochondrial proteins ATP6, CO1, Cytb, ND2, and ND5 [74]. Concerning scavenging enzymes, RSV is able to upregulate glutathione peroxidase, catalase [76], and MnSOD [77] expression in endothelial cells, in a SIRT1-dependent manner [77, 78].

### 3.3. Effects of Curcumin on Mitochondrial ROS

Curcumin displays antioxidant and cytoprotective effects on several cell types, including hepatoma cell lines, retinal epithelial cells, astrocytes, and spinal cord astrocytes [71, 78–80]. Curcumin exerts its antioxidant properties through direct and indirect mechanisms. Indeed, Cur is an effective scavenger of free radicals such as hydroxyl radical (OH^*∙*^), O_2_^−^, nitric oxide (NO), H_2_O_2_, and peroxynitrite [81–86]. Concerning indirect mechanisms, Cur is able to upregulate cytoprotective cell response by modulating the expression of genes encoding antioxidant proteins, such as superoxide dismutase (SOD), catalase (CAT), heme oxygenase-1 (HO-1), or proteins that replenish the glutathione pool such as glutathione reductase (GR), glutathione peroxidase (GPx), and GST [87–90]. As in the case of RSV, the upregulation of these genes is induced by the Cur-mediated transactivation of Nrf2 and has been demonstrated in several* in vitro* cell models [91, 92] as well as* in vivo* ones [93, 94]. The antioxidant and cytoprotective effects of Cur have been proven to be beneficial also* in vivo.* Similarly to Qu, Cur protects cardiac cells from ischemia reperfusion (I/R) damage by reducing oxidative stress and by helping cells to maintain intact mitochondrial functions [81]; in an* in vivo* model of chronic kidney disease, Cur displayed cardioprotective effects that were mediated by diminished ROS production and by the maintenance of mitochondrial functions, such as OXPHOS [95, 96].

Cur can also have a cytoprotective effect against toxic compounds able to generate ROS and to cause lipid peroxidation and DNA damage, such as potassium dichromate (K_2_Cr_2_O_7_). Indeed, several studies have shown that Cu pretreatment has a protective role against toxicity of K_2_Cr_2_O_7_ for kidney, liver, and male reproductive system [97–99].* In vivo*, Cur prevents the decrease in body weight caused by K_2_Cr_2_O_7_ and increases liver weight and liver/body ratio and exerts a protective effect against oxidative damage to liver tissue, by preventing the decrease of hepatic antioxidant enzymes caused by K_2_Cr_2_O_7_. These effects appear to be mediated by a protective effect on mitochondria. Indeed, studies on isolated organelles showed that Cur reduces mitochondrial dysfunction by preventing the reduction of complex I activity and the opening of the PTP induced by K_2_Cr_2_O_7_. This preventive activity blocks the release of cyt c, likely inhibiting mitochondrial-induced apoptosis [99].

A similar, protective effect has been demonstrated in rats treated with indomethacin, a potent ROS inducer: administration of Cur prevented oxidative stress and maintained mitochondrial functions in cells from colon [100].

## 4. Effects of Natural Compounds on Mitochondrial-Mediated Apoptosis and Mitophagy and on Mitochondrial Biogenesis

In the last twenty years, the effects of natural compounds on apoptosis have been subject of huge investigations, mainly aimed at identifying molecules able to selectively cause death of cancer cells [101]. However, it must be noted that the results of this type of studies are often difficult to interpret, because of the enormous variety of* in vitro* and* in vivo* model used, the dose dependency of the effects of many compounds, and the capability of the same compounds to exert a prosurvival effect in cancer cells, by favouring mitochondrial biogenesis and cell proliferation. The systematic reanalysis of this plethora of studies goes further beyond the purpose of this review; in this paragraph, we will summarize the direct and indirect mechanisms by which QU, RSV, and Cur modulate mitochondria-mediated apoptosis (summarized in Table 2) or, conversely, increase mitochondrial biogenesis.

### 4.1. Quercetin, Mitochondrial Biogenesis, and Apoptosis

Data concerning the effects of Qu on mitochondrial biogenesis are quite controversial. In HepG2 cells, Qu induces mitochondrial biogenesis through activation of HO-1 [102]. Conversely,* in vivo* data obtained on mice or rats gave opposite results, depending on the experimental design and the cell type taken into account. Higher expression of PPAR-gamma, cytochrome c (cyt c) oxidase, and citrate synthase were noted; furthermore, increased mitochondrial biogenesis was accompanied by higher levels of mtDNA [103]. Conversely, other authors have shown that muscle mitochondrial biogenesis should be attributed exclusively to exercise and that Qu supplementation in the diet had negligible effect on mitochondria in mice fed with high-fat diet [104]. The combination of oral Qu supplementation and exercise has been shown to prevent brain mitochondrial biogenesis [105].

The capability of Qu to trigger apoptosis* via* mitochondrial pathway has been shown in a variety of cell models [38, 106–111]. It is particularly interesting to observe that, in some cases, Qu causes cell death in cancer cells, but not in the parental, nonmalignant cells [112].

Qu is able to trigger mitochondria-mediated apoptosis both by direct and indirect mechanisms [11]. Concerning direct mechanisms, Qu induces loss of mitochondrial membrane potential (MMP), release of cytochrome c from mitochondria, and the subsequent activation of caspase-3 and caspase-7 [106, 108]. Experiments on isolated mitochondria from rat liver have shown that Qu causes the release of cyt c by inhibiting adenine nucleotide translocase (ANT), which in turn determines the opening of the permeability transition pore (PTP), through a cyclosporin A insensitive mechanism [38]. In several cell models, the capability to induce apoptosis appears to be correlated with the capability of Qu to deplete GSH, an event that precedes loss of MMP, phosphatidylserine exposure, decrease of mitochondrial mass, and subsequent cell death [11, 112].

Qu can also favour apoptosis by modulating the expression of pro- and antiapoptotic proteins belonging to the Bcl-2 family. In particular, Qu upregulates Bax and Bak and downregulates Bcl-2 and Bcl-xL [108, 110], thus determining the multimerization of Bax to the mitochondrial membrane.

Another indirect mechanism by which Qu exerts a proapoptotic activity is the generation of ROS. As stated above, Qu can increase intracellular ROS levels, as Qu radicals can be formed after peroxidase-catalyzed oxidation in order to scavenge reactive peroxyl radicals [113]. In some conditions, Qu generates enough ROS to trigger free radical-induced apoptosis, through the activation of the AMPK1/ASK1/p38 pathway [114]. Accordingly, the generation of ROS determines the subsequent activation of AMPKalpha1 and ASK1, which are accompanied by activation of p38 and recruitment of caspases [115–117].

### 4.2. Resveratrol, Mitochondrial Biogenesis, and Apoptosis

Several studies indicate that RSV can have some beneficial effects on mitochondrial biogenesis and activity [41]. In particular, it has been shown that RSV supplementation in the diet of rodents is associated with an increase in mtDNA content and of protein levels of mitochondrial transcription factor A (Tfam) and PGC-1*α*; this increase is mirrored by an increase in oxygen consumption and in the activity of respiratory and lipid-oxidizing mitochondrial enzymes [42–44, 118]. Concerning mitochondrial biogenesis, RSV-stimulating effects are mediated by a mechanism involving three main actors, namely, PGC1a, SIRT1, and AMPK [41, 119]. As mentioned above, SIRT1 is activated in cells exposed to RSV and other polyphenols, including flavonoids, butein, catechins, and Cur [120]. Whether RSV acts directly on SIRT1 or its action is indirect is still matter of debate. While some authors have shown that RSV can directly act on Sir2, the yeast ortholog of human SIRT1 in* Saccharomyces cerevisiae*, [121] others did not evidence any direct effect and ascribed the observed phenomenon to technical problems [122, 123]. Accumulating data now indicate that the effects on SIRT1 are mediated by AMPK activation. Finally, it must be noted that a recent study casted some doubts on the effects of RSV on mitochondrial biogenesis, at least in muscle cells [124].

RSV has the capability to induce apoptosis in different manners. At high concentration (100 *μ*M), RSV induces apoptosis in breast cancer cell lines [125], by provoking rapid depolarization of mitochondria, release of Ca^2+^ from the ER, followed by opening of PTP, release of cyt c, and activation of caspases; xenograft experiments further confirm that RSV treatment inhibits breast cancer growth [125]. The same effect has been observed in hepatocarcinoma cells [126]. RSV acts as an antagonist of antiapoptotic proteins, therefore favouring the induction of apoptosis in cancer cells. In particular, it induces the upregulation of p21 in a p53-independent manner, which in turn determines cell cycle arrest, depletion of the antiapoptotic protein survivin, and sensitization to TRAIL-mediated apoptosis [127]. RSV also suppresses the expression of the antiapoptotic proteins Bcl-xL, Mcl-1, and Bcl-2 in different human cancer cell lines [128, 129]; in U937 cells, this effect is due to the suppression of constitutively active NF-kB, through RSV-mediated inhibition of IkB. The ectopic overexpression of Bcl-2 attenuates RSV proapoptotic effect, confirming the proapoptotic effect of this molecule through the downregulation of antiapoptotic genes [130]. Furthermore, RSV can favour apoptosis by increasing the expression of the proapoptotic protein Bax [131, 132] or by inducing oligomerization of Bax on mitochondria [133].

### 4.3. Curcumin, Mitochondrial Biogenesis, and Apoptosis

Data concerning Cur effects on mitochondrial biogenesis are scarce and mainly obtained by* in vivo* studies [134–137]. In hepatocytes isolated from rats, Cur treatment increases mtDNA copy number and upregulates transcriptional factors that regulate mitochondrial biogenesis, including PGC1*α*, Nrf1, and Tfam [134].* In vivo* studies on rats subjected to I/R injury further confirm that Cur increases mitochondrial biogenesis. Indeed, Cur pretreatment reverts the reduction in Nrf-1 and Tfam and in the number of mitochondria observed with I/R and helps in reducing infarct volume and in maintaining neuron functionality, in a dose-dependent manner [138].

As mentioned above, the anticancer properties of Cur rely on its capacity to inhibit proliferation and induce cancer cell death. Many studies, performed on different human and murine cell types, indicate that Cur, like Qu and RSV, can have both proapoptotic and cytoprotective effects, depending on the dose or cell model used [139].

The mechanisms by which Cur induces cancer cell death are not clearly defined and are likely mediated by different pathways; nevertheless, the crucial role of mitochondria-mediated apoptosis is well established in different cell models. At high concentration (80 *μ*M), Cur has a prooxidant activity, as it leads to increased levels of O_2_^−∙^ and causes cell death in human colon cancer cells in a p53-independent manner [140]. The crucial role of mitochondria in Cur-mediated apoptosis has been demonstrated in isolated rat liver organelles: in this model, Cur induces an increase in the membrane permeability, resulting in swelling, loss of membrane potential, and inhibition of ATP synthesis; this effect is mediated by PTP opening [141]. In human glioblastoma cells, treatment with Cur at relatively low concentrations (25–50 *μ*M) causes release of* cyt* c and AIF from mitochondria and subsequent cell death [142]; a similar effect has been observed in colorectal cancer cells [143]. Like other proapoptotic phytochemicals, Cur targets proliferative cells more efficiently than differentiated cells. For instance, Cur induces a rapid decrease in MMP and the release of cyt c followed by cell death in growing murine neural 2a (N2a) cells, but not in differentiated N2a cells [144].

The proapoptotic effects of Cur are also exerted in an indirect manner, through the upregulation of proapoptotic proteins located in mitochondria. In human breast cancer cells, Cur induces apoptosis* via* a p53-dependent pathway in which Bax is upregulated and renders cells more prone to apoptosis [145]. The crucial role of proapoptotic proteins of Bcl-2 family, such as Bax, Bak, Bim, and Bid in Cur-mediated apoptosis, has been further confirmed in other cell models [143, 146, 147]. In colorectal cancer cells, Cur sensitizes cells to apoptosis by upregulating of Bax, Bak, Bim, and Bid, as well as Apaf-1, and by inducing the oligomerization of Bax, which in turn favours the release of cyt c from mitochondria [143]. Cur can also favour apoptosis by downregulating antiapoptotic proteins, such as Bcl-2 [137], or by downregulating NF-kappaB, which in turn determines the downregulation of both Bcl-2 and Bcl-X_L_ [148].

Finally, Cur exerts an indirect proapoptotic activity by damaging mtDNA. Indeed, the prooxidant activity of Cur at high concentrations damages both mtDNA and nDNA in HepG2 cells, but with a more dramatic effect on mtDNA [135, 136]. Such damage causes impairment of OXPHOS, reduces ATP synthesis, and renders cells more prone to cell death. The observation that mtDNA-depleted cells are resistant to Cur-induced apoptosis further confirms the essential role of mtDNA in the sensitivity to cell death [137].

## 5. Concluding Remarks

Natural compounds display a panoply of effects on mitochondria, affecting virtually every function correlated with the biology of the organelle. Data concerning differential effects on cancerous and normal, nontransformed cells are particularly interesting for possible, therapeutic use of these molecules as chemotherapeutics or chemopreventers. Furthermore, as aging in mammals is associated with mitochondrial oxidative stress in virtually every tissue [149–153], the use of these molecules, and particularly RSV, as antiaging agents is considered of particular interest [154–157]. Nevertheless, several problems must be solved before thinking of a wide, systematic use of these natural compounds in the clinical practice.

First, contradictory data have been obtained in different cell models, and these discrepancies need urgent clarification, particularly to understand which are the doses that display beneficiary on mitochondria, without causing collateral, dangerous effects. Second, rigorous studies on large cohorts of subjects are urgently needed to clearly define the daily intake and bioavailability of these natural compounds. Indeed, the actual clinical potential of these molecules cannot be fully established until proper protocols providing optimal bioavailability to ensure sufficient tissue distribution are established. Third, studies on natural compounds of vegetal origin are usually focused on few, well-known molecules or on herbal extracts whose composition is barely known and often nonstandardized. The expansion of the array of molecules analysed in depth as Qu, RSV, or Cur will open new perspectives in the modulation of mitochondrial functions related with the onset of human diseases.

## Figures and Tables

**Table 1:** Direct and indirect effects of Qu, RSV, and Cur on mitochondrial ROS.

Molecule	Effects on mitochondrial ROS	Model used	Doses	Time of treatment	References
*Quercetin *					
Direct effects	Scavenging of O_2_^∙−^	Cell-free system; CHO cells	10–200 *μ*M	Up to 24 hours	[49]
	Scavenging of H_2_O_2_	Cell-free system; CHO cells	10–200 *μ*M	Up to 24 hours	[49]
Direct effects as prooxidant	Production of semiquinone radical and Qu-quinone, which depletes GSH	Cell-free system; CHO, U937, THP-1, HL-60, and NB4 cells	10–200 *μ*M	Up to 24 hours	[49, 51–53]
Indirect effects on antioxidant systems	Inhibition of TrxR	Cell-free system; A549 cells	25–100 *μ*M	24 hours	[56]

*Resveratrol *					
Direct effects as antioxidant	Scavenging of O_2_^∙−^	U937, K562, HepG2 MCF-7, NHEK cells; RAW 264.7, JB6 cells; Wistar-Kyoto rats	0–150 *μ*M	Up to 48 hours	[65–67]
	Scavenging of ^*∙*^OH	U937, K562 HepG2 MCF-7, NHEK cells	0–150 *μ*M	Up to 48 hours	[65, 66]
	Scavenging of H_2_O_2_	N9 microglial cells, C6 astroglial cells	25–100 *μ*M	0–600 secs	[64]
Indirect effects on antioxidant systems	Upregulates glutathione peroxidase and catalase	Rat coronary endothelial cells	1–100 *μ*M	48 hours	[76]
	Upregulates MnSOD	Human coronary endothelial cells	1–10 *μ*M	48 hours	[77]
	Activates Nrf2 mediated antioxidant response	Normal human epidermal keratinocytes	20–100 *μ*M	16 hours	[73]

*Curcumin *					
Direct effects as antioxidant	Scavenging of O_2_^∙−^	Cell-free system; heart homogenate from Wistar rats	0–200 *μ*M	48 hours	[81, 82]
	Scavenging of ^*∙*^OH	Rat L-6 myoblasts	0–4 *μ*M	30 mins	[84]
	Scavenging of H_2_O_2_	Cell-free system; Rat L-6 myoblasts	15–45 *μ*g/mL; 0–4 *μ*M	30 mins	[82, 84]
	Scavenging of ONOO^−^				[85]
	Scavenging of NO^*∙*^	Cell-free system; G108-15 neuroblastoma-glioma cells	1–25 *μ*M		[86]
	Scavenging of ROO^*∙*^	Rat L-6 myoblasts	0–4 *μ*M	30 mins	[84]
Indirect effects on antioxidant systems	Upregulation of antioxidant enzymes (SOD, CAT, and HO-1)	C6 rat glioma cells; rat cerebellar granule neurons; ECV304 human endothelial cells	0–100 *μ*M	Up to 48 hours	[87–89]
	Replenishment of glutathione pool via upregulation of GR, GPx, and GST	Chick liver	74 mg/kg	Up to 21 days	[90]

**Table 2:** Direct and in direct effects of Qu, RSV, and Cur on mitochondria-mediated apoptosis.

Molecule	Effects on mitochondrial-mediated apoptosis	Model used	Doses tested	Time of treatment	References
*Quercetin *					
Direct proapoptotic effect	Loss of MMP, followed by release of cyt c	U937 cells; MDA-MB-231 cells	0–300 *μ*M	Up to 24 hours	[106, 108]
	Inhibition of ANT and opening of PTP, followed by release of cyt c	Isolated mitochondria from rat kidney	0–50 *μ*M	Up to 10 minutes	[38]
	Depletion of GSH, followed by loss of MMP and cyt c release	U937 cells, human peripheral blood mononuclear cells	0–100 *μ*M	Up to 24 hours	[11, 112]
Indirect proapoptotic effects	Upregulation of Bax and Bak and downregulation of Bcl-2 and Bcl-xL	HepG2 cells	0–200 *μ*M	Up to 72 hours	[108, 110]
	Activation of AMPK1/ASK1/p38 pathway through ROS increase	MCF-7 breast cancer cells; HCT116 and HT-29 cells	0–400 *μ*M	Up to 24 hours	[109–111]

*Resveratrol *					
Direct proapoptotic effects	Loss of MMP, opening of PTP, and release of cyt c	MCF-7, MDA-MB-231 cells, and HepG2 cells	0–200 *μ*M	Up to 48 hours	[125, 126]
Indirect proapoptotic effects	Upregulation of p21 mediated by p53	Neuroblastoma (SHEP, GIMEN, and LAN5), medulloblastoma (PSFK), glioblastoma (U373MG, A172), melanoma (SK-Mel, Colo38), pancreatic (MiaPaCa2), prostate (LNCaP), and breast carcinoma (MCF-7) cells	0–100 *μ*M	Up to 24 hours	[127]
	Downregulation of Bcl-xL, Mcl-1, and Bcl-2	Ramos and Raji, TIB-196 and CCL-155 B cell lines	0–200 *μ*M	Up to 24 hours	[128, 129]
	Upregulation and oligomerization of Bax	HCT116 cells; F344 rats	0–100 *μ*M; 200 *μ*g/kg body wt/day	32 hours; three months	[131, 132]

*Curcumin *					
Direct proapoptotic effects	Increase of O_2_^∙−^, followed by increase in mitochondrial permeability and release of cyt c	HCT-116 and HT-29 cells	0–160 *μ*M	Up to 72 hours	[140]
	PTP opening, followed by mitochondrial swelling and increase in permeability	Isolated mitochondria from rat liver	0–20 *μ*M	Up to 15 minutes	[141]
	Release of cyt c and AIF	T98G, PC3, LNCaP, MDA-MB23, Jurkat cells, and immortalized human fibroblasts	15–25 *μ*M	Up to 24 hours	[142, 143]
Indirect proapoptotic effects	Upregulation of Bax via a p53-dependent pathway	MCF-7 cells	10 *μ*M	Up to 48 hours	[145]
	Upregulation of Bax, Bak, Bim, Bid, and Apaf-1	HCT-116, PC3, LNCaP, MDA-MB23, Jurkat cells, immortalized human fibroblasts, and embryonic fibroblasts	0–50 *μ*M	Up to 72 hours	[143, 146, 147]
	Downregulation of Bcl-2 and Bcl-X_L_	HepG2, U266, and MM.1 cells	0–50 *μ*M	Up to 16 hours	[137, 148]
	Damage of mtDNA, which impairs mitochondrial functions	HepG2, HT1080, and HEK293T cells	0–40 *μ*M	Up to 24 hours	[135, 136]
